# 3D biodegradable scaffolds of polycaprolactone with silicate-containing hydroxyapatite microparticles for bone tissue engineering: high-resolution tomography and *in vitro* study

**DOI:** 10.1038/s41598-018-27097-7

**Published:** 2018-06-11

**Authors:** Svetlana Shkarina, Roman Shkarin, Venera Weinhardt, Elizaveta Melnik, Gabriele Vacun, Petra J. Kluger, Kateryna Loza, Matthias Epple, Sergei I. Ivlev, Tilo Baumbach, Maria A. Surmeneva, Roman A. Surmenev

**Affiliations:** 1 Research Center “Physical Materials Science and Composite Materials”, National Research Tomsk Polytechnic University, 634050 Tomsk, Russian Federation; 2 Laboratory for Applications of Synchrotron Radiation, Karlsruhe Institute of Technology, Eggenstein-Leopoldshafen, Germany; 3 Institute for Applied Computer Science, Karlsruhe Institute of Technology, Karlsruhe, Germany; 4 Centre for Organismal Studies, University of Heidelberg, Heidelberg, Germany; 5 Institute for Photon Science and Synchrotron Radiation, Karlsruhe Institute of Technology, Eggenstein-Leopoldshafen, Germany; 6 Fraunhofer Institute for Interfacial Engineering and Biotechnology IGB, Stuttgart, Germany; 7 Fachbereich Chemie, Philipps-Universität Marburg, Marburg, Germany; 8 Inorganic Chemistry and Center for Nanointegration Duisburg-Essen (CeNIDE), University of Duisburg-Essen, Essen, Germany; 9 Reutlingen University, Reutlingen, Germany

## Abstract

To date, special interest has been paid to composite scaffolds based on polymers enriched with hydroxyapatite (HA). However, the role of HA containing different trace elements such as silicate in the structure of a polymer scaffold has not yet been fully explored. Here, we report the potential use of silicate-containing hydroxyapatite (SiHA) microparticles and microparticle aggregates in the predominant range from 2.23 to 12.40 µm in combination with polycaprolactone (PCL) as a hybrid scaffold with randomly oriented and well-aligned microfibers for regeneration of bone tissue. Chemical and mechanical properties of the developed 3D scaffolds were investigated with XRD, FTIR, EDX and tensile testing. Furthermore, the internal structure and surface morphology of the scaffolds were analyzed using synchrotron X-ray µCT and SEM. Upon culturing human mesenchymal stem cells (hMSC) on PCL-SiHA scaffolds, we found that both SiHA inclusion and microfiber orientation affected cell adhesion. The best hMSCs viability was revealed at 10 day for the PCL-SiHA scaffolds with well-aligned structure (~82%). It is expected that novel hybrid scaffolds of PCL will improve tissue ingrowth *in vivo* due to hydrophilic SiHA microparticles in combination with randomly oriented and well-aligned PCL microfibers, which mimic the structure of extracellular matrix of bone tissue.

## Introduction

Bone is a rigid, complex form of connective tissue of the skeleton that protects vital organs from damage and provides support for the entire body. It is composed of a fibrous organic matrix impregnated with inorganic minerals, such as calcium and phosphate, which promote the hardness and toughness of tissue^1^. Throughout life, bone is constantly created and renewed through a process called remodeling. However, this process can fail in various bone disorders, including bone fractures and diseases. Traditional methods of bone treatment available today include various graft types, which have several disadvantages, including the potential to cause an immune reaction and the risk of disease transmission^2^.

As an alternative, novel principles of tissue engineering can be applied to develop artificial bone three-dimensional (3D) scaffolds, which have recently attracted much interest^3^. Some challenges associated with scaffold fabrication are that it must possess appropriate chemical and mechanical properties and highly porous interconnected structure with variable pore-size distribution, which supports cell attachment, proliferation and differentiation by mimicking the extracellular matrix (ECM) of natural bone tissue.

A variety of polymers and ceramics have been developed as biomaterials for the substitution of bone tissues^4^. Many researchers have shown that polycaprolactone (PCL) is a polymer that can be successfully applied for the fabrication of artificial 3D scaffolds; it is bioresorbable with an appropriate mechanical elasticity suited for long-term bone implantation applications^5,6^. More than 70% of the inorganic composition of bone is primarily composed of HA, which has frequently been used in the bioengineering of bone tissues^7,8^. Several recent studies have focused on the fabrication of hybrid PCL scaffolds enriched with HA nanoparticles^9,10^. Earlier the presence of HA in the PCL matrix showed successful attachment, proliferation and osteogenic differentiation of various cell types, compared to pure PCL scaffolds^11,12^. For enhancement of scaffold’s bioactivity, it is possible to combine PCL with HA, modified with different trace elements. For instance, the addition of zinc-doped (Zn) HA to the PCL/chitosan nanocomposite scaffold has shown enhanced cell attachment and proliferation during *in vitro* tests^13^.

On the other hand, silicate (Si) containing HA could be another alternative to pure HA that stimulated biological activity during bone tissue formation more than pure HA^14,15^. In the 1970s, it was demonstrated that mineralization requires a minimum of soluble silicon^16^. Thus, the combination of the properties of HA and Si allowed the development of a new SiHA composite, resulting in a patent, “Silicon-Substituted Apatite and Process for the Preparation”^17^. In a recent review, enhanced biocompatibility and positive effect of pure SiHA on bone formation during *in vitro*/*in vivo* experiments and clinical applications was discussed^18^. In addition, the results of *in vivo* tests of HA and SiHA powders have demonstrated the induced rate and amount of bone apposition for SiHA bioceramics^19^. Recently, thin coatings of SiHA were deposited on titanium substrates by RF-magnetron sputtering^20^. According to *in vitro* studies, the SiHA coatings showed enhanced cell adhesion^21^. Study of the porous SiHA scaffolds with varying silicon contents in a rabbit model by Hing *et al*. indicated that after one week of implantation, the best cellular penetration and bone ingrowth were observed for material with a silicon content of 0.8 wt.%^22^.

The design and structure of biomaterials, which able to mimic natural ECM, should be taken into account. For scaffold fabrication, many studies employ an electrospinning technique, which is a simple and versatile method for generating 3D fibrous structures with a high surface-area-to-volume ratio close to the ECM with an interconnected pore structure and variable fiber diameter^23^. Efforts to mimic the natural ECM have led researchers to create special designs for collectors to form fibers with different degrees of alignment for specific orientation and guided proliferation of cell culture, which is highly preferred^24,25^. This approach can help to control mechanical properties, due to better fiber-packing over scaffolds with decreasing porosity.

While SiHA is widely applied in different studies as a coating for metallic substrates or the fabrication of entire scaffolds devoted to the substitution of bone tissues^26,27^, it is not used in the electrospinning process for fabrication of polymer 3D scaffolds.

To explore the potential for cellular penetration and bone in-growth into polymer scaffolds, we have designed and fabricated 3D composite scaffolds with a different fiber orientation containing SiHA particles. A different fiber orientation could help to mimic ECM; however, SiHA particles may improve the bioactivity and osteogenic potential of PCL-based scaffolds.

To achieve this objective, we prepared specifically tailored PCL and PCL-SiHA scaffolds with randomly oriented and well-aligned structures using an electrospinning process. As the internal structure of hybrid scaffolds includes weakly (polymer) and highly (SiHA particles) absorbing materials, for complete 3D morphological characterization, high-resolution synchrotron micro-computed tomography (µCT) was used. Synchrotron X-ray µCT is a powerful technique for non-destructive testing that can be used to characterize the complex scaffold structure presented in this study. The major advantage of this method is the ability to study an object with high penetration depth, high spatial resolution and a large field of view in a 3D space. Due to the noninvasive character of this technique, it is possible to avoid any physical interaction, compared to other conventional imaging techniques, such as optical and electron microscopy techniques, which require special sample preparation.

## Results and Discussion

### Chemical composition of electrospun scaffolds

**Figure 1:**
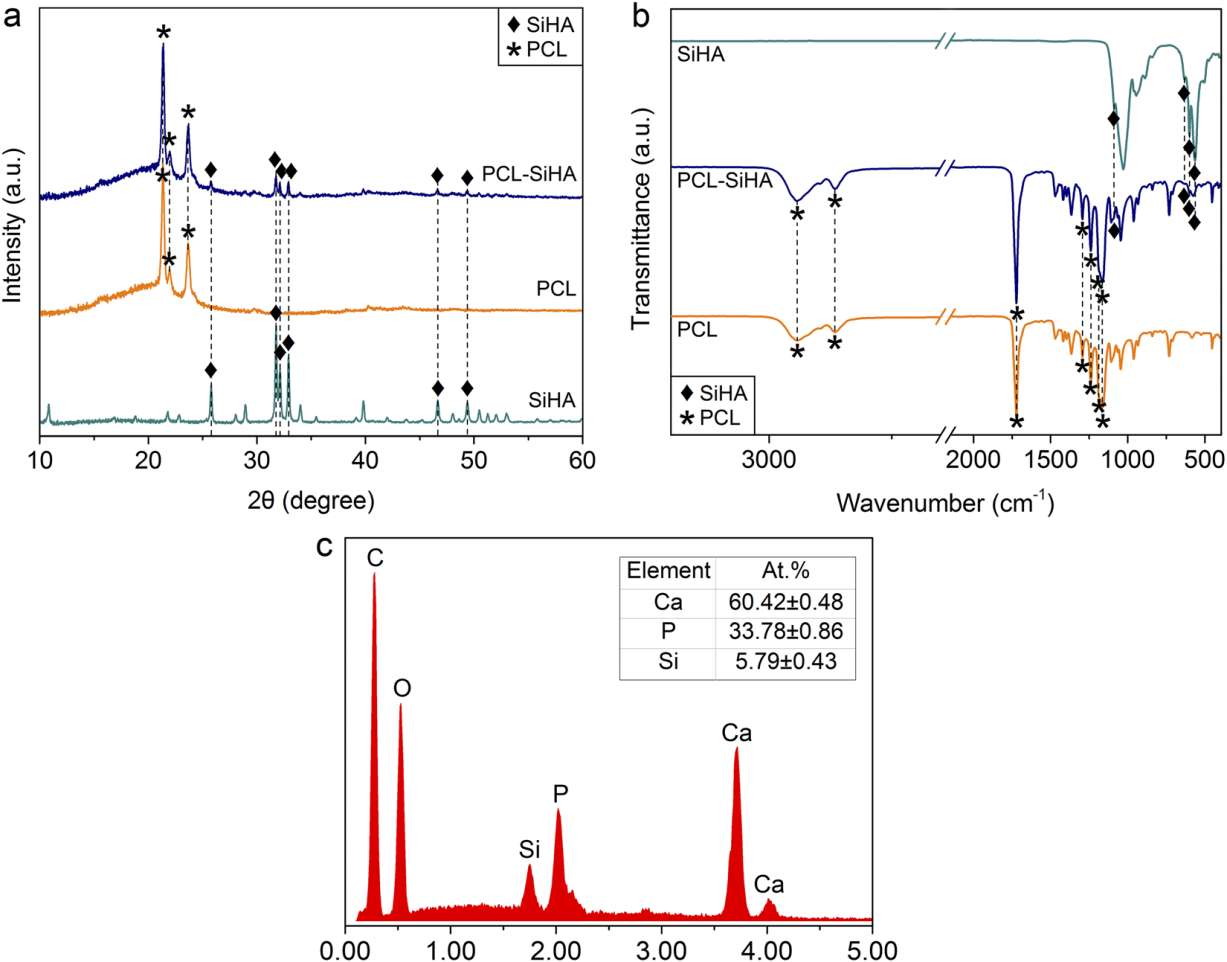
**(a)** X-ray diffraction patterns; **(b)** FTIR spectra of PCL, PCL-SiHA scaffolds and SiHA powder. The results are presented for samples with randomly oriented structure. **(с)** EDX spectrum revealing elemental composition of rPCL-SiHA scaffold with predominant calcium and phosphorus peaks. The insert label shows the quantitative results on the content of P, Ca and Si atoms in the rPCL-SiHA and wPCL-SiHA scaffolds.

The nonwoven scaffolds with an interconnected porous structure were successfully fabricated using the electrospinning process. The presence of SiHA has been proven with XRD and IR analyses, as shown in Fig. 1a,b. As the polymer solution used for the synthesis of randomly oriented and well-aligned structures was the same, the XRD patterns and FTIR spectra showed no significant differences between the polymer structures. In the XRD pattern (Fig. 1a), the strongest reflections of semicrystalline PCL polymer were detected at 21.36° (110) and 23.68° (overlapping 200, 013, 112, 104 reflections), in good agreement with the literature data^28^. The broad amorphous halo in the low-angle region (2θ ≤ 30°) of the pattern confirms the presence of the amorphous phase. The diffraction pattern also contains the reflection of SiHA (the most intensive peaks at 25.77°, 31.74°, 32.15°, 32.89°, 34.06°, 46.69°, 49.42°), which can be indexed as the hexagonal primitive cell with the following lattice parameters: a = 9.4218(7), c = 6.9063(10) Å, V = 530.94(9) Å^3^ at 293 K, in good agreement with the crystal data for unsubstituted HA^29^. This means that after incorporation into the PCL-SiHA composite, SiHA retains its crystalline nature, as deduced from the presence of the SiHA diffraction maxima in the PCL-SiHA powder diffraction pattern. Using the Scherrer equation, the mean crystallite size of SiHA as incorporated into the PCL matrix was estimated as 44 nm.

Supplementary Fig. S1 shows the Le Bail profile fit for the PCL and SiHA phases. The above unit cell parameters were used to refine the SiHA phase. For the PCL phase, well-known cell parameters in the space group P2_1_2_1_2_1_ were used^28^, which was refined to the following values: a = 7.521(3), b = 4.9894(10), c = 17.164(6) Å, V = 644.08(40) Å^3^.

Figure 1b shows the FTIR spectra for PCL, PCL-SiHA scaffolds and SiHA powder. The typical characteristic peaks of PCL appear in the ranges of 1000–1800 cm^−1^ and 2800–3000 cm^−1^. All peaks are in good correspondence with the published data^30^. The strong band belonging to the carbonyl stretching mode at 1720 cm^−1^ is well resolved. The two peaks at 2864 cm^−1^ and 2942 cm^−1^ correspond to the symmetric and asymmetric stretching of the CH_2_ group, and the two peaks at 1239 cm^−1^ and 1162 cm^−1^ correspond to the symmetric and asymmetric stretching of the C-O-C group. Furthermore, the bands at 1193 cm^−1^ and 1293 cm^−1^ are related to O-C-O stretching, and C-O and C-C stretching in the crystalline phase, respectively^31^. The characteristic peaks of PO_4_^3−^ attributed to SiHA are found at 563 cm^−1^, 603 cm^−1^, 632 cm^−1^, 887 cm^−1^ and 1087 cm^−1^ ^15,32,33^. The results obtained with the powder XRD and FTIR spectroscopy confirm the successful incorporation of SiHA into the PCL-SiHA hybrid scaffolds.

Also, the presence of SiHA in the PCL matrix was confirmed by EDX (Fig. 1c). The value of Ca/(P + Si) ratio for rPCL-SiHA and wPCL-SiHA scaffolds was calculated to be 1.52 ± 0.03, which is close to stoichiometric Ca/P ratio in HA (1.67) and is in the acceptable range found in the literature for use in biomedical application^34,35^. The difference between Ca/(P + Si) ratio of SiHA precursor powder and that of Ca/(P + Si) in microparticles embedded into the scaffolds may be due to the porosity of the samples as well as an inhomogeneous distribution of the agglomerated SiHA particles within the scaffolds. Moreover, EDX is only a qualitative technique, which is also surface roughness sensitive, some deviations can be observed when porous and pore-free samples of the same composition are studied, which makes the results different to compare. It is believed that HA with the stoichiometric Ca/P ratio of 1.67 possesses the best mechanical properties compared to nonstoichiometric HA^36^, which may affect dissolution properties during interaction with the surrounding biological media. Seo *et al*. stated that dissolution of HA was more distinct in the samples with Ca/P ratio less or more than stoichiometric^37^.

### Characterization of the structure and morphology with synchrotron µCT

The morphology and structure of fibers play an important role in controlling the adhesion and proliferation of cells^38^. In the case of composite scaffolds, the addition of Si-HA to the polymer solution can cause the distortion of fibers. The phenomenon of changes in the fiber diameter was observed by Metwally *et al*., who produced PCL scaffolds with pure HA and calcium carbonate microparticles during electrospinning^39^.

**Figure 2:**
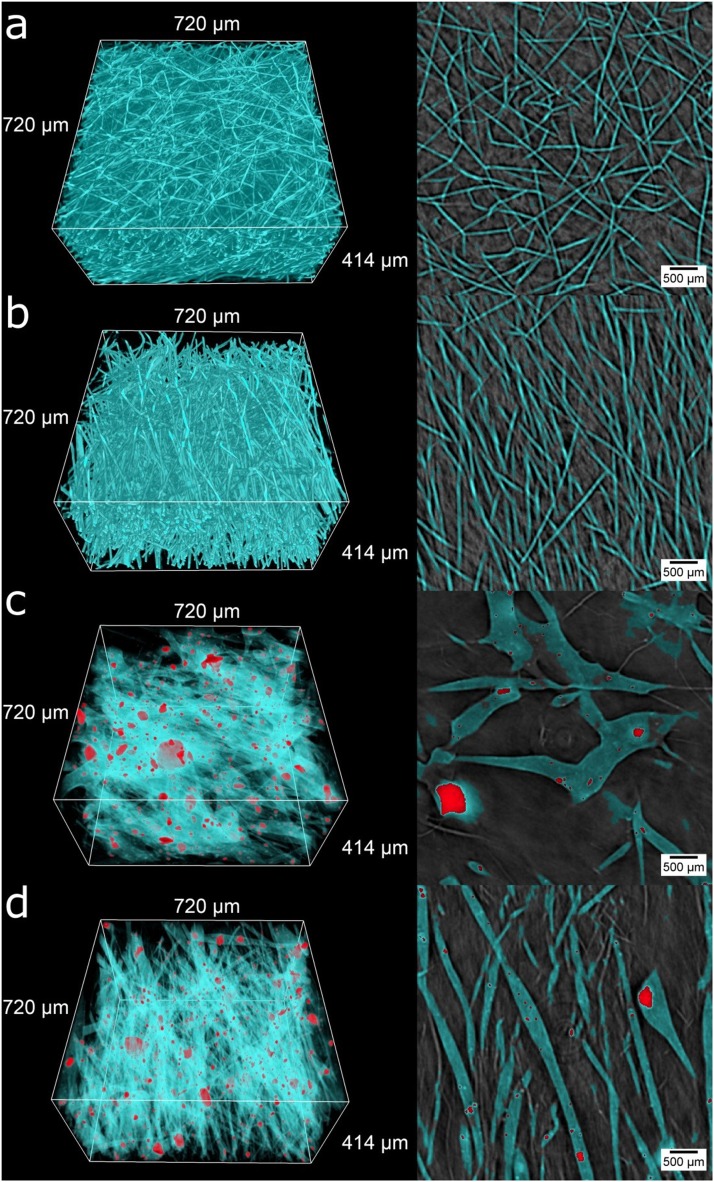
X-ray µCT-based visualization of polymer PCL and hybrid PCL-SiHA scaffolds: 3D rendering in left column and transverse sections through sample height in right column for: **(a)** rPCL, **(b)** wPCL, **(c)** rPCL-SiHA and **(d)** wPCL-SiHA. Cyan – fibers, red– microparticles and their aggregates.

Here, we use 3D reconstructed volumes of synchrotron µCT, presented in Fig. 2 (cyan – fibers, red – microparticles and their aggregates). The detailed visual observations of the 3D scaffold structure are presented in the supplementary information (Movies S1–4). For a more representative view, the regions of interest (ROI) of 400 × 400 × 230 pixels, which correspond to 720 × 720 × 414 µm^3^, were extracted. Observations of the surface morphology demonstrated that all of the obtained PCL and PCL-SiHA scaffolds were fabricated with the desired fiber morphology, namely, a randomly oriented and well-aligned fiber orientation.

Similar to previous studies^40,41^, we detected changes in the fiber morphology for samples containing SiHA microparticles. In the case of rPCL-SiHA and wPCL-SiHA samples, a beaded rough surface morphology with some very large polymer structures was observed. The intensive agglomeration of SiHA microparticles occurred over the entire volume. The largest aggregates are seemingly embedded within an even larger polymer structure. Such a change in morphology may result from the change in electrostatic forces, as Si inclusions are non-conductive and/or increase in viscosity with respect to pure PCL.

**Figure 3:**
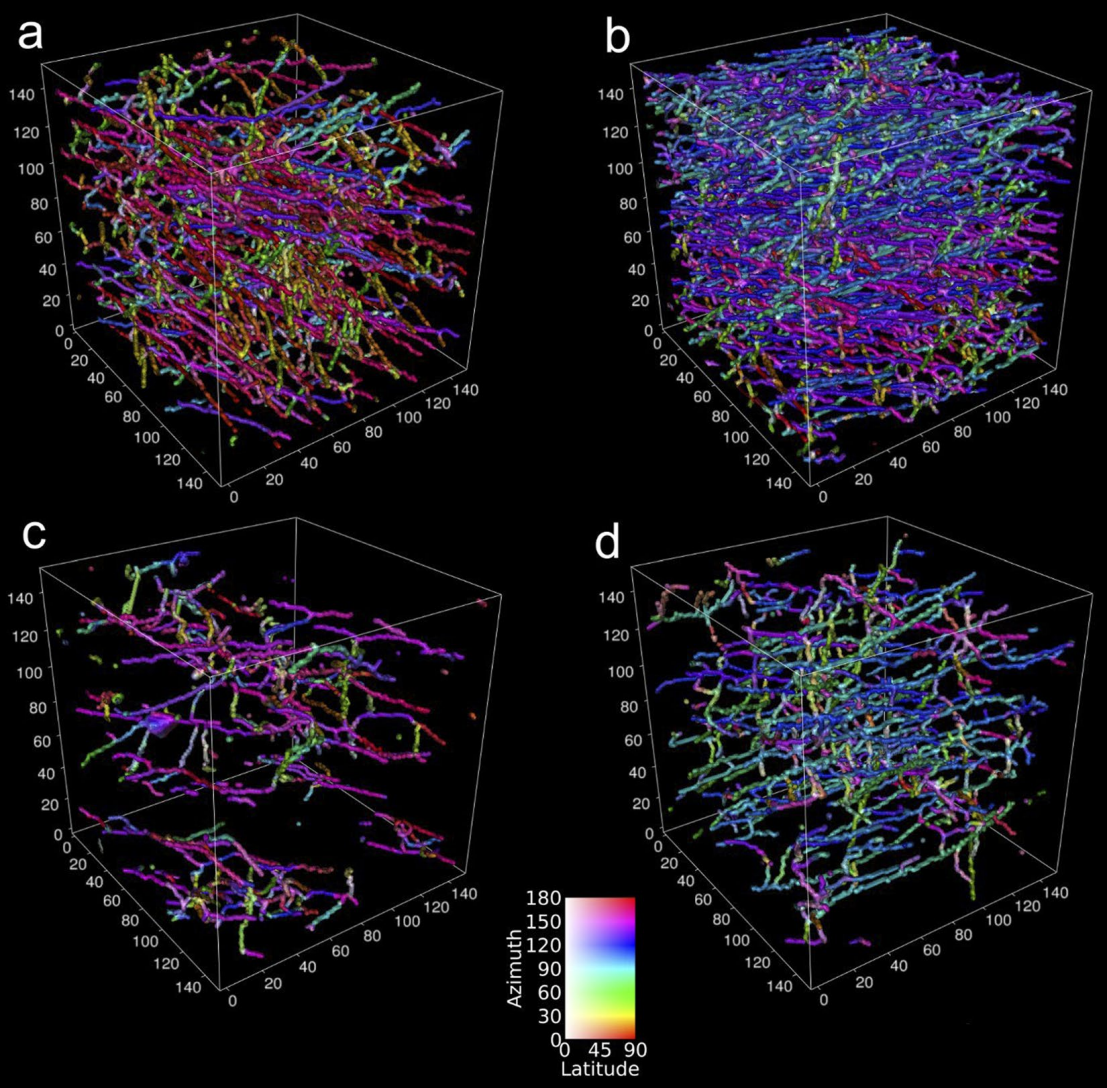
Visualization of fiber orientation for 3D scaffolds: **(a)** rPCL, **(b)** wPCL, **(c)** rPCL-SiHA and **(d)** wPCL-SiHA.

Supplementary Fig. S2 shows azimuthal and latitudinal fiber orientation histograms. In the azimuthal direction (Supplementary Fig. S2a), samples with a well-aligned structure have more fibers with the predominant orientation in the 75–100° angle range. In the case of scaffolds with a randomly oriented structure, there is a predominant directionality of fibers due to the type of collector (rotating collector), but in lower amounts, namely, for rPCL in the range of 80–160° and for rPCL-SiHA in the range of approximately 20–60° and 120–180°. Notably, in the latitudinal orientation, all samples show similar results for angles from 50° to 90°, based on layer-by-layer fiber deposition during electrospinning (Supplementary Fig. S2b). Full 3D analysis of fiber orientation is presented in Fig. 3. This 3D analysis grants an immediate visual comparison between randomly oriented and well-aligned fiber orientations. Fibers aligned in a similar direction are represented by the same color. Of particular interest, fibers in wPCL change their preferential direction depending on the height of the sample, demonstrating a difference in the layer deposition during electrospinning. The samples with SiHA inclusions have fewer or larger fibers, but they still preserve a random and aligned orientation. It is clearly visible that in the case of scaffolds with an aligned structure, most fibers lay in the same plane compared to scaffolds with a randomly oriented structure, where fibers are chaotically distributed in the sample volume.

**Figure 4:**
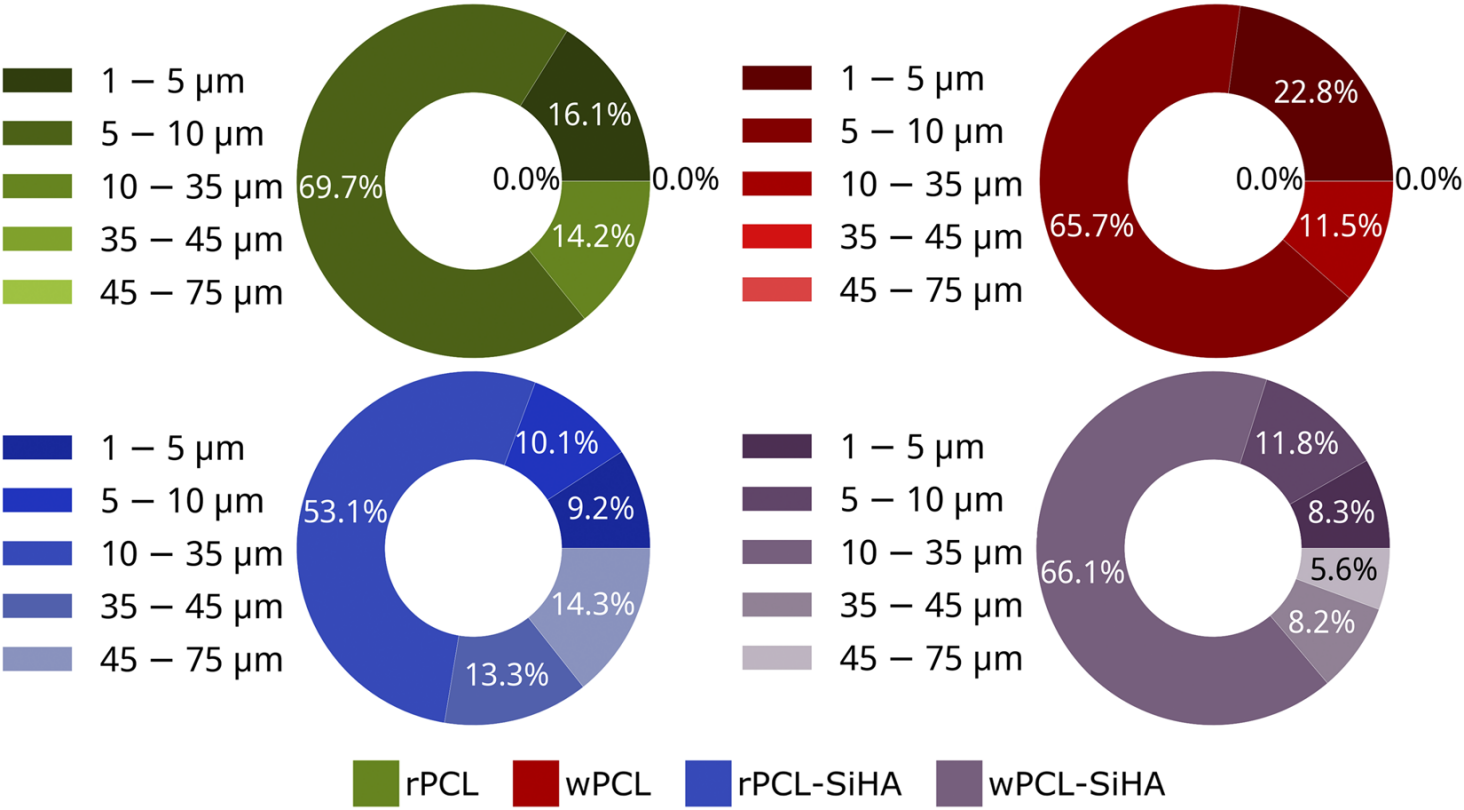
X-ray µCT-based analysis of fiber diameter calculated for 3D scaffolds.

For the detailed study of fiber diameter (Fig. 4), the largest fibers were in the range of 35–75 µm for hybrid scaffolds, and the number of fibers with the size in the range of 10–25 µm was higher. It was also observed that the diameter of fibers for wPCL and wPCL-SiHA scaffolds decreased compared to rPCL and rPCL-SiHA scaffolds. Pie charts showed that the fiber diameter of wPCL increased in the range from 1 to 5 µm for 6.7% and decreased from 10 to 35 µm for 2.7%, relative to the rPCL sample. We also observed the same trend for samples with SiHA microparticles, where the fiber diameter of wPCL-SiHA increased in the smallest diameter range from 1 µm to 10 µm for 0.8%, from 10 µm to 25 µm to 14.4% and decreased from 25 µm to 35 µm for 1.3%, from 35 µm to 45 µm for 5.1%, and from 45 µm to 75 µm for 8.7%, relative to the rPCL-SiHA sample. This can be explained due to extra stretching of fibers at higher collection speeds.

**Figure 5:**
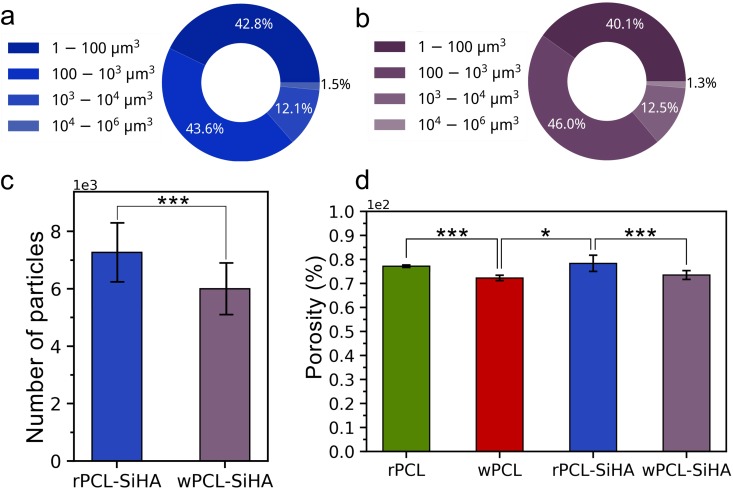
X-ray µCT-based analysis. Pie charts showing the percentage of a total number of microparticles and microparticle aggregates in each size region for **(a)** rPCL-SiHA and **(b)** wPCL-SiHA; **(c)** histogram displaying the total number of microparticles and aggregates presented in composites and **(d)** histogram showing the porosity of polymer and hybrid scaffolds (*p < 0.05, **p < 0.1, ***p < 0.3, N.S. with p > 0.8).

The results of the analysis of the size distribution of the SiHA microparticles and microparticle aggregates in rPCL-SiHA and wPCL-SiHA are presented in Fig. 5a,b. The most commonly detected SiHA microparticle aggregates were in the range from 5.83 µm^3^ to 1000 µm^3^, corresponding to an approximate diameter from 2.23 µm to 12.4 µm, assuming the microparticle aggregates can be represented as a sphere. Larger microparticle aggregates were detected from 1000 µm^3^ to 1000000 µm^3^, corresponding to an approximate diameter from 12.4 µm to 124 µm. However, their number comprised the smallest percentage in the whole volume of the scaffolds and was not higher than 13.6% for rPCL-SiHA and 13.8% for wPCL-SiHA. Importantly, during synchrotron µCT examination, microparticles less than 5.83 µm^3^ could not be detected using automatic segmentation algorithms. However, application of the high-resolution µCT presented in this study makes it possible to estimate the internal features of scaffolds as small as ~1.8 µm. A similar number of microparticles and their aggregates was observed for both hybrid types of scaffolds, and no statistical significance was recognized (Fig. 5c).

The porosity of the samples that contain SiHA reveals slightly increased values of 78.36 ± 3.35% and 73.50 ± 1.87% for rPCL-SiHA and wPCL-SiHA, respectively, compared to the values of 77.14 ± 0.49% and 72.27 ± 1.14%, for rPCL and wPCL samples, respectively (Fig. 5d). According to these results, SiHA microparticles and their aggregates contribute to changes in porosity, and this fact is of great importance for the optimization of scaffold design, which may aid in the enhanced attachment and proliferation of cells inside the scaffold^42^.

**Figure 6:**
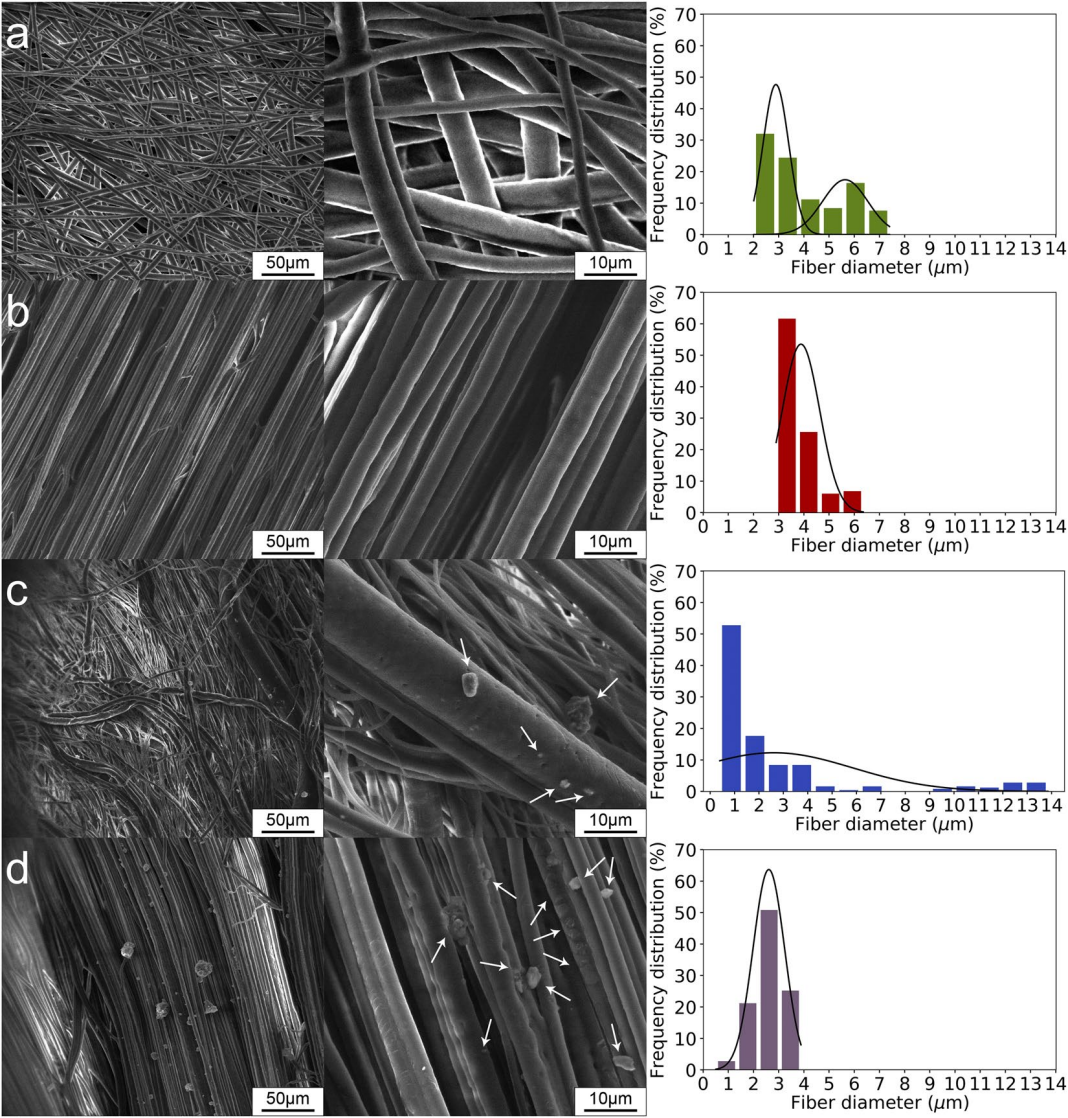
SEM micrographs of scaffolds: **(a)** rPCL, **(b)** wPCL, **(c)** rPCL-SiHA and **(d)** wPCL-SiHA. Arrows indicate presence of SiHA.

X-ray µCT provided inherently quantitative information on the fiber diameter, fiber orientation and distribution of SiHA inclusions. However, the large field of view available with this method comes at the cost of spatial resolution and does not allow a precise analysis of microparticles and fibers less than ~1.8 µm. For this reason, we additionally studied the surface of the scaffolds using SEM, and the results are presented in Fig. 6. Similar to the µCT results, SEM images illustrate the embedding of large fibers in the case of rPCL-SiHA and wPCL-SiHA samples (Fig. 6c,d). Most of the scaffolds are characterized by the presence of fibers at the micron level, especially for the rPCL-SiHA scaffold. In the case of rPCL scaffolds, a bimodal fiber diameter distribution was observed, which can be associated with the formation of satellite drops and can be controlled by the viscoelastic properties of the polymer solution^43^. In the case of rPCL-SiHA, the presence of microparticles and their aggregates leads to a unimodal diameter distribution.

### Mechanical testing

**Figure 7:**
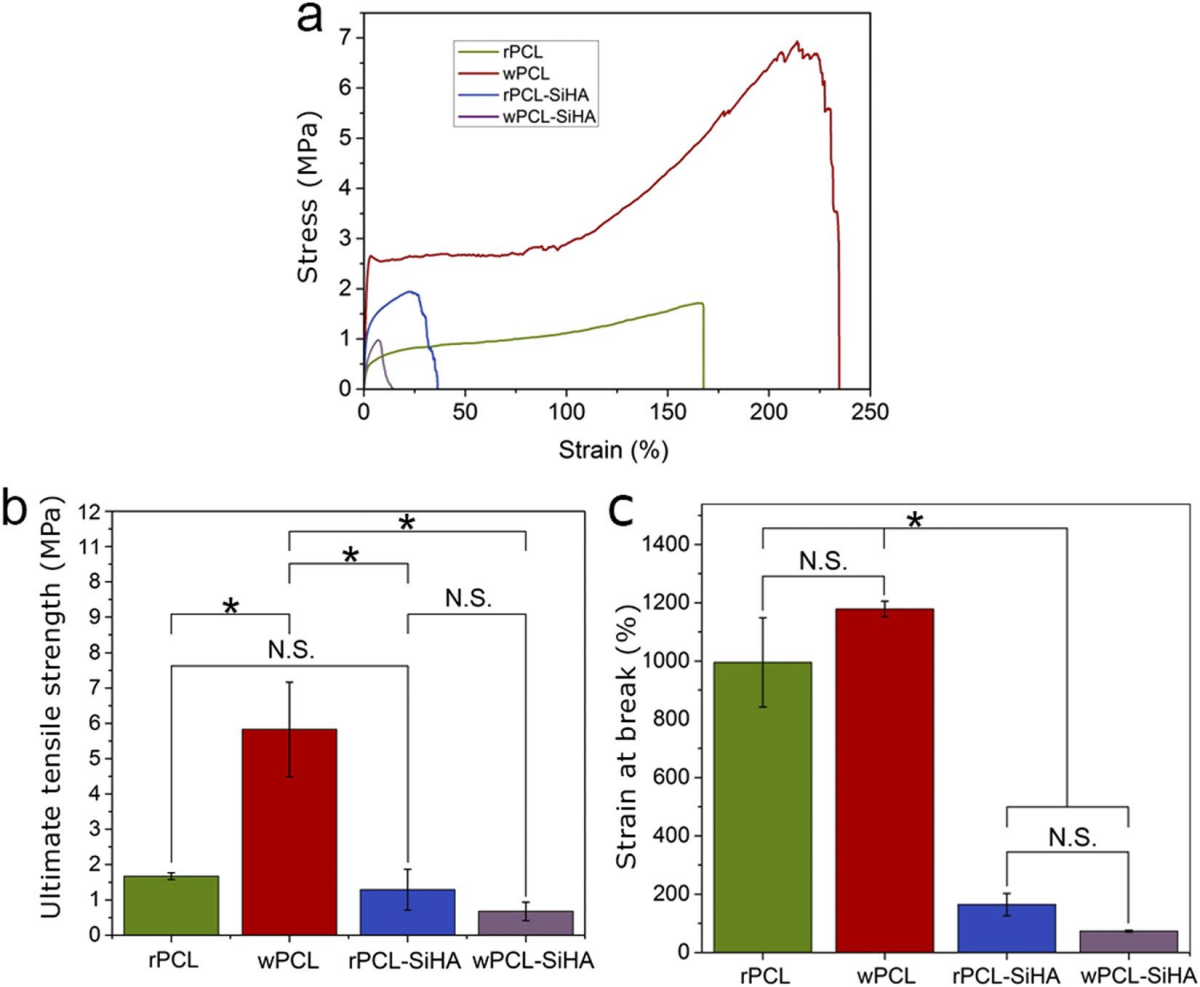
Tensile testing of electrospun PCL and hybrid scaffolds: **(a)** stress-strain curve, **(b)** ultimate tensile strength, **(c)** strain at break (*p < 0.05, N.S. with p > 0.8).

The mechanical properties of the PCL and PCL-SiHA scaffolds are determined by several factors, including the fiber structure, distribution, and size of fillers, as well as the interfacial bonding strength. The presence of microparticles and their aggregates in the structure of polymer fibers may lead to an interfacial lack of adhesion between two materials, which induces the ability of the matrix to transversely relocate stress across the interface^44^ and to the formation of pores and debonding of the fibers due to a lack of adhesion^45^. These facts may cause a decrease in mechanical properties of scaffolds^40,46^. Thus, we analyzed the mechanical properties of PCL-SiHA scaffolds using tensile testing (Fig. 7). It was revealed that in the case of pure PCL scaffolds, the tensile strength (Fig. 7b) was significantly larger for wPCL than for the rest of the samples due to parallel fiber alignment in one direction.

We observed that in the case of hybrid scaffolds, the addition of SiHA microparticles and microparticle aggregates reduced the mechanical properties for both rPCL-SiHA and wPCL-SiHA scaffolds. For comparison, the ultimate tensile strength was 1.29 ± 0.58 MPa and 0.67 ± 0.26 MPa for rPCL-SiHA and wPCL-SiHA, respectively, whereas for rPCL and wPCL, the values were 1.67 ± 0.1 MPa and 5.83 ± 1.34 MPa (Fig. 7b). In addition, the elongation at break of rPCL was 995.23 ± 153.16%, which decreased to 164.42 ± 38.30% for rPCL-SiHA. The same trend was observed for samples with a well-aligned structure, but the values significantly changed (p < 0.05) from 1178.24 ± 26.54% for wPCL to 73.14 ± 2.90% for wPCL-SiHA (Fig. 7c). This supports the hypothesis of weak bonding between SiHA microparticle aggregates and the polymer matrix due to weak van der Waals interactions^46^. The large aggregates of SiHA in the polymer scaffold lead to failure at a lower stress. According to the tomography results, the large aggregates presented in the hybrid scaffolds are up to 124 µm in size and thus can cause failure at lower stress. Furthermore, we assume that changes in the mechanical properties can also be caused by the increase in porosity, which means that there is less material compared to pure polymer scaffolds that are able to resist mechanical stress.

Extensive studies have attempted to characterize the tensile properties of various human bones. Thus, Goldstein *et al*. demonstrated that the mechanical properties of fresh frozen trabecular bone varied by up to two orders (strength 1–13 MPa) of magnitude depending on the load-bearing areas in bone tissue^47^. Lindahl also revealed the range of mechanical strength for the tibia, in the frames of 0.2–6.7 MPa for the male group^48^. Behrens *et al*., in their study of fresh frozen cancellous bone, showed that the strength of the bones varied from 1.8 to 63.6 MPa^49^.

Based on the mechanical testing, designing a multilayered PCL and PCL-SiHA scaffold with functionally graded composition and structure from a composite solution with randomly oriented and well-aligned structures (tensile strength of 5 MPa) can be very promising for bone tissue engineering applications.

### Wettability

**Figure 8:**
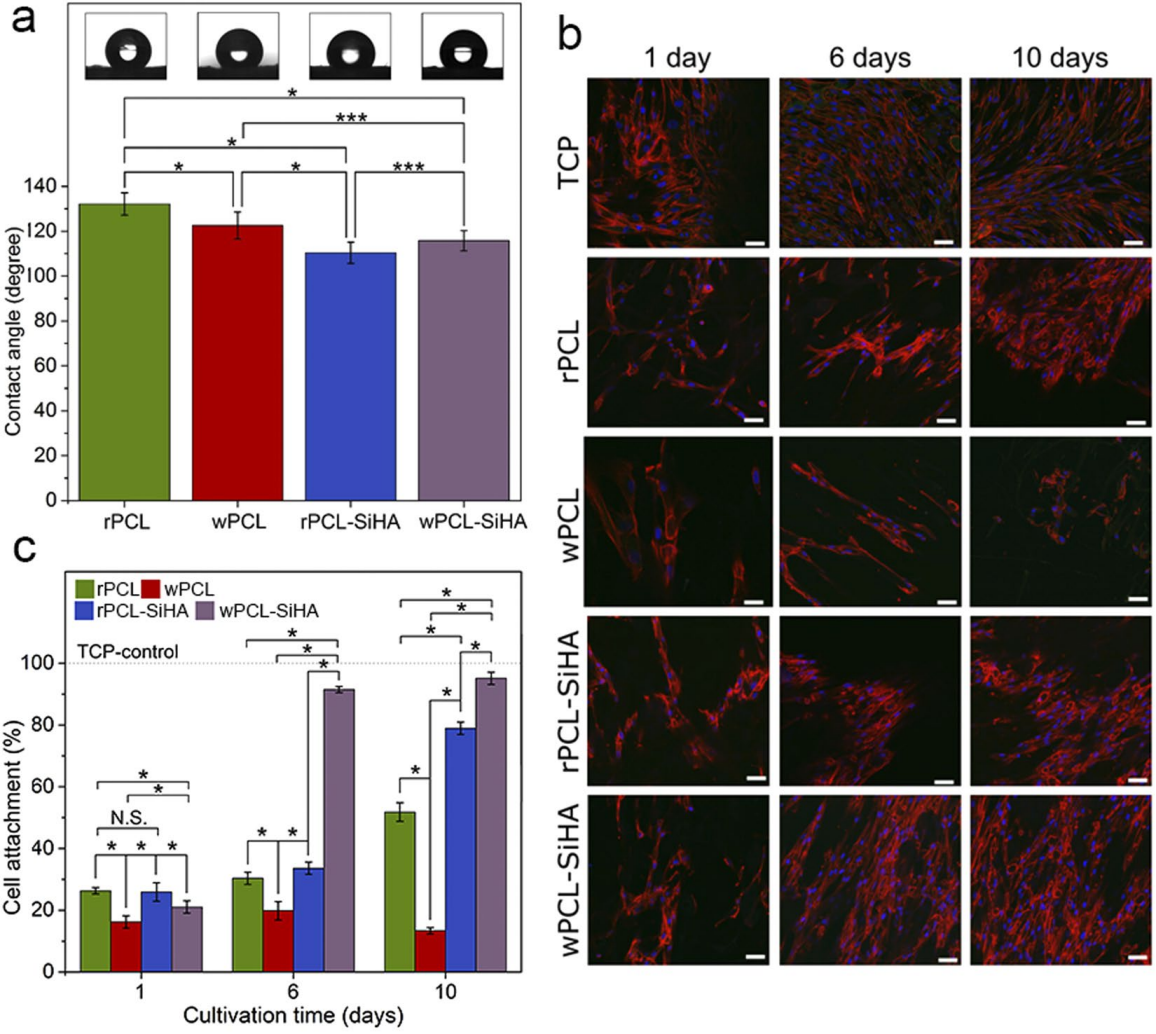
**(a)** Water contact angles of electrospun PCL polymer and PCL-SiHA hybrid scaffolds (*p < 0.05, **p < 0.1, ***p < 0.3, N.S. with p > 0.8); **(b)** cell adhesion of hMSCs cultured on electrospun scaffolds was evaluated by fluorescence staining for actin cytoskeleton (red) and nuclei (blue), TCP was used as a control, scale bar = 50 µm, in all images; **(c)** percentage of hMSCs cells attached to PCL and PCL-SiHA scaffolds at 1, 6 and 10 days post-seeding (*p < 0.05, **p < 0.1, ***p < 0.3, N.S. with p > 0.8).

Previous reports showed that the PCL polymer has a contact angle of approximately 70° ^50,51^. To understand the influence of SiHA agglomerates on the wetting ability of the electrospun scaffolds, contact angle measurements were performed. According to Fig. 8a, the contact angle values for pure PCL scaffolds vary from 132.13 ± 4.95° (rPCL) to 122.54 ± 5.97° (wPCL) for randomly oriented and well-aligned structures, respectively. Possible porous structures of the polymer scaffolds act as a rough surface, which leads to an increase in the contact angle value compared to the flat surface^50^. The reduced contact angle for the wPCL scaffolds can be explained by the spreading of a water droplet along the fiber orientation.

A decreased contact angle in the case of composite scaffolds was previously observed by Mi *et al*. for HA-containing hybrid scaffolds^40^ and by Xu *et al*. for electrospun poly(L-lactide)-grafted HA/poly(L-lactide) nanocomposite fibers^52^. Similar to their findings, the addition of SiHA particles to the PCL scaffolds resulted in a decrease in the contact angle values. The difference between pure scaffolds and polymer scaffolds containing SiHA with randomly oriented and well-aligned fibers was 21.75 ± 0.35° and 6.79 ± 1.5°, respectively. SiHA is hydrophilic, which can cause the rapid absorption of water by SiHA microparticles/aggregates.

### Cell adhesion and proliferation analysis

The viability of cells is a crucial issue for the clinical use of tissue-engineered 3D scaffolds. In the literature *in vitro* studies demonstrated enhanced adhesion and proliferation of hMSCs on pure SiHA compared with pure HA^18^. For this reason in our study, we analysed the influence of SiHA in polymer PCL matrix with different fiber orientation by performing adhesion tests with hMSCs, which have the possibility to differentiate into the bone cells (osteoblasts). In Fig. 8b, the CLSM images of cell adhesion and proliferation on the randomly oriented (rPCL and rPCL-SiHA) and well-aligned (wPCL and wPCL-SiHA) fibrous scaffolds after 1, 6 and 10 days of culture under static conditions are shown. At day 1, the hMSCs showed similar proliferation activity on the surface of all scaffolds. However, at day 6, the number of cells on the composite rPCL-SiHA scaffolds increased 3.23 ± 0.22% compared to rPCL. Furthermore, on the wPCL-SiHA scaffolds, the number of cells significantly increased by 71.66 ± 5.73% compared to wPCL and almost formed a fusion on the surface of the fibrous mesh compared to the other scaffolds. At the same time point, other scaffolds have shown no significant difference in cell number. At day 10, the largest cell number was demonstrated for wPCL-SiHA, and the number of cells increased compared to wPCL for 81.78 ± 4.92%. The rPCL-SiHA scaffolds showed a significant increase in the number of cells of 27.12 ± 1.6% compared to rPCL, which was quite close to wPCL-SiHA.

Comparison of the changes in cell number between day 1 and day 10 showed that the cell number increased by 53.03 ± 4.24% and 74.09 ± 5.53% for the rPCL-SiHA and wPCL-SiHA scaffolds, respectively. In the case of pure rPCL, the number of cells also slightly increased by 25.51 ± 2.04%. However, the wPCL scaffolds showed poor cells adhesion, and the number of cells did not increase but actually decreased by 2.83 ± 0.19%.

It is evident from Fig. 8c that cell proliferation is significantly higher on hybrid rPCL-SiHA and wPCL-SiHA scaffolds, possibly due to the hybrid composition of the scaffolds or increased porosity due to addition of SiHA inclusions.

The poor cell activity on pure PCL scaffolds may be due to the hydrophobic nature of the polymer. In fact, the presence of SiHA decreased the hydrophobicity of samples and increased surface roughness and porosity, which could promote a better spreading of cells compared to the smoother, less porous and more hydrophobic surface of pure PCL fibers^53^. Interestingly, contrary to our expectations based on the literature^54^, the wPCL scaffold showed poor results over the entire culture period. At day 10, the number of cells decreased dramatically compared to other scaffolds. The results obtained from *in vitro* tests indicated that hybrid scaffolds were more suitable substrates than pure PCL scaffolds for cell attachment and proliferation.

In conclusion, this paper reports the fabrication and characterization of PCL scaffolds for regeneration of bone tissue with randomly oriented and well-aligned fiber structures. To improve cellular penetration and bone in-growth, we embedded SiHA microparticles and microparticle aggregates into the polymer 3D scaffolds’ fiber structure. XRD and FTIR analyses confirmed the presence of SiHA in the composition of hybrid scaffolds. The X-ray µCT provided 3D quantitative data on the structure of scaffolds, such as the fiber diameter and orientation, porosity, microparticle/aggregate size, and the number distributed over the entire sample with high contrast and spatial resolution. According to µCT analysis, the most frequently detected SiHA microparticles and their aggregates were in the range from 2.23 µm to 12.4 µm (assuming that they can be represented as a sphere), which caused the maximum effect in changes of the fiber morphology and mechanical properties. SEM served as a complementary tool in the morphological study to analyze the diameter of fibers and particles in the micrometer range. From a mechanical point of view, 3D scaffolds without SiHA were more tensile-resistant, especially wPCL scaffolds. However, the adhesion and proliferation of hMSCs was increased for hybrid scaffolds, especially the wPCL-SiHA scaffold. In comparison, cell viability at day 6 was slightly increased for the hybrid rPCL-SiHA scaffold in ~3% and significantly increased in ~72% for wPCL-SiHA compared to pure rPCL and wPCL scaffolds. At day 10, cell viability increased up to ~27% for composite scaffolds with randomly oriented structure compared to pure rPCL, and up to ~82% for composite scaffolds with a well-aligned structure compared to pure wPCL. From the above we may conclude that not only the presence of SiHA microparticles and microparticle aggregates, but the microfiber orientation of the composite scaffolds in the random and aligned directions have significantly affected the efficiency of the cell-scaffold interaction. The bioactive properties of hybrid scaffolds containing different combinations of cation- and anion-substituted HA and polymers, including piezoelectric properties, may be the most interesting field for future pre-clinical studies.

## Methods

### Scaffold fabrication

Precursor powder of SiHA (Ca_10_(PO_4_)_5.2_(SiO_4_)_0.8_(OH)_1.2_) was purchased from the Institute of Solid State Chemistry and Mechanochemistry SB RAS (Novosibirsk, Russia). Pellets of PCL (number-average molecular weight = 80,000 Da) and chloroform solvent (chemically pure) were obtained from Sigma-Aldrich (St. Louis, MO, USA).

Fibrous scaffolds with randomly oriented (rPCL) and well-aligned (wPCL) structures were produced via electrospinning. PCL pellets were dissolved in chloroform at a concentration of 9% (w/v) and homogenized using a vibrating shaker (Multi-Reax, Heidolph, Germany) for 8 hours, followed by ultrasonication for 30 min. To produce hybrid scaffolds with randomly oriented and well-aligned structures, the PCL solution was mixed with 10 wt.% of Si-HA (rPCL-SiHA, wPCL-SiHA). Then, solutions were placed in 10-ml plastic syringes with a 20-gauge stainless steel needle. A solution feeding rate of 2 ml/h was used for the syringe pump (AJ-5803, Angel Electronic Equipment Co., China). A high-voltage power supply (Hurletron HVG 30–5/H, Eltex Elektrostatik GmbH., Germany) connected to the needle provided a voltage of 17 kV to deposit fibers on a grounded collector, which represents a metallic mandrel situated 7 cm from the needle tip. Randomly oriented and well-aligned structures were obtained on a rotating collector at a speed of 600 and 1000 rpm, respectively. Electrospinning was performed at ambient temperature with a duration of 1 hour for all samples.

### Scaffold Characterization

Scaffold morphology was characterized with a Quanta 400 scanning electron microscope (FEI, Netherlands) equipped with energy dispersive X-ray detector (EDX) for elemental analysis, including the presence of SiHA microparticles in case of the hybrid scaffolds. Hybrid scaffolds were cut into squares with a sharp blade, sputter-coated with gold and examined at an accelerating voltage of 10 kV. Average values and standard deviations of the fiber diameter were determined by measuring the size of 100 fibers from four SEM images.

X-ray diffraction (XRD) patterns were recorded on a STOE STADI MP diffractometer (STOE&Cie GmbH., Germany) using Cu-K_α1_ radiation (λ = 1.540598 nm, operating at 40 kV and 40 mA), a germanium monochromator, and a Mythen1K detector. The diffraction patterns were collected within the 2θ range of 10°–60°, with an angle step of 0.8° and irradiation time of 10 s per step. The diffraction data were handled using Jana2006 software^55^.

The mean crystallite size of SiHA was determined using the Scherrer equation after fitting the XRD data with a pseudo-Voigt profile function in Jana2006. The diffraction maximum at 2θ = 32.89° was chosen for the calculation, due to its good resolution and an absence of interference with the adjacent maxima.

The infrared spectra of the samples were recorded using a Tensor 37 FTIR spectrometer (Bruker Optics, Germany) and the OPUS software package (Bruker Optics, Germany). The spectra were collected in the 4000–400 cm^−1^ region, with 2 cm^−1^ resolution accumulating in a total of 100 scans.

The electrospun nanofiber scaffolds were mechanically tested using an Instron 3343 tensile testing machine (Instron Ltd., USA) at room temperature. Scaffolds were cut into rectangular strips with average dimensions of 5 × 20 × 0.5 mm. The specimens were mounted in the vertical direction between two mechanical gripping units, leaving a 4-mm gauge length, and the cross-head speed was set at 40 mm/min. Data from the stress versus strain curves were recorded, and the tensile stress at maximal load was obtained. For each scaffold, several experiments were performed and averaged to determine the tensile properties of the scaffolds.

A water contact angle test was performed at room temperature using droplet shape analysis using an OCA 15 Plus apparatus (Data Physics Instruments GmbH, Germany) and SCA20 software (Data Physics Instruments GmbH, Germany). Ten measurements were performed at different locations using deionized water (2 µL) droplets, and the average value of the contact angles was used for the calculations.

### Synchrotron µCT imaging and data analysis

High-resolution µCT was performed at the micro-imaging station using a bending magnet source at the Institute for Photon Science and Synchrotron Radiation of the Karlsruhe Institute of Technology (KIT, Karlsruhe, Germany)^56^. A detailed description of the station setup is presented elsewhere^57^. The monochromatic beam was used in conjunction with an sCMOS Camera (sensor size 5.5 megapixels, 6.5 μm physical pixel size), and a 200-μm-thick Lu_3_Al_5_O_12_ scintillator and BAM macroscope 3.6x microscope optics allowed for a spatial resolution of approximately 1.8 µm to give a visual field of view of 4.6 × 3.9 mm. A propagation distance of approximately 20 mm was set between the samples and detector. The samples were rotated with a step of 0.24^o^ and were exposed for 1 sec with 12 keV X-rays. All projections were normalized to the dark signal of the camera and a reference beam. Tomographic reconstruction was performed using a filtered back projection algorithm implemented in the UFO framework^58^. Analysis of the reconstructed datasets consists of quantification of the particle/fiber size distribution, as well as determination of the porosity parameter of each sample and the orientation distribution of the fiber structures. Before dataset analysis, each slice of the reconstructed data was filtered with a median filter of radius 1.8 µm. It was segmented using a threshold-based maximum entropy method, which is implemented with the Numpy and SciPy packages for Python programming language^59^. The analyzed volumes were 800 × 800 × 350 pixels, corresponding to 1440 × 1440 × 630 µm^3^. The segmentation process produces a binary dataset where only particles have non-zero values. When particles were segmented, the obtained binary data were analyzed with the connected component algorithm implemented in the Scikit-Image package. The algorithm isolates non-zero regions in 3D binary data for microparticles if they do not touch each other at faces or edges^60^.

Orientation and diameter analysis of the 3D fibers was performed at points of a skeleton extracted from the segmented fibers with help of the quanfima package^61^. At each point of the skeleton, the second-order structure tensor was calculated in a 3D local window with a size of 57.6 µm. Eigenvalues and eigenvectors were derived from each tensor with the linear algebra module of the SciPy package^59^. Fiber orientation was determined with the eigenvector, which corresponds to the minimal eigenvalue. A fiber can be modeled as a vector *ρ* from the origin of the spherical coordinate system, oriented at *θ* and *φ* angles corresponding to azimuth and latitude angles, respectively (Supplementary Fig. S3).

Porosity analysis was performed by calculating the ratio of the number of voxels in the segmented volume composed of microparticles, fibers or both to the total number of voxels in the volume. Amira 5.4.1 software (FEI, Visualization Sciences Group) was used for 3D visualization. Two-dimensional (2D) plotting was performed with the Matplotlib package^62^.

### In vitro cell seeding

To investigate the electrospun 3D scaffold’s capability to support cell adhesion and proliferation, human mesenchymal stem cells (hMSCs) were purchased from Lonza (Walkersville, USA). Before experiments, samples were cut into 15 mm × 15 mm squares and placed in 6-well culture plates, which were sterilized with 70% ethanol for 10 minutes, rinsed once with DPBS (Sigma-Aldrich, Munich, Germany) and air-dried. A total of 80 µl of hMSCs in a mesenchymal stem cell medium (mesenchymal stem cell medium, serum-free enhanced, Pelobiotech, Plannegg/Martinsried, Germany) supplemented with 50 units/ml of penicillin and 50 µg/ml of streptomycin was seeded onto a 1 cm² area per sample. As a control tissue, a culture polystyrene (TCP) dish was chosen. The cells were left to attach for 3 h in a humidified atmosphere (37 °C, 5% CO_2_). Then, each sample was immersed in 4 ml of human stem cell medium and cultured for 1, 6 and 10 days.

The interaction of cells with scaffolds was investigated using confocal laser scanning microscopy (CLSM). For CLSM analysis, cells grown on scaffolds were washed in DPBS (Biochrom GmbH, Germany) and fixed with Histofix (Roth, Germany) for 10 min. The fixed samples were washed twice and permeabilized for 10 min in a Triton X100 solution (0.5% in DPBS). After two washing steps, the samples were incubated with 1:50 diluted phalloidin Alexa Fluor® 546 (Life Technologies, Darmstadt, Germany) in an antibody diluent (Dako, Hamburg, Germany) for 1 h at room temperature. The samples were rinsed three times with DPBS, followed by an incubation step with 1:50 diluted Vinculin-FITC conjugated antibody (Sigma-Aldrich, Germany) in antibody diluent for 1 h. After three washing steps with DPBS, the nuclei were stained with DAPI (Sigma-Aldrich, Germany) at a concentration of 1 µg/ml in DPBS for 15 min. The samples were rinsed twice with DPBS and analyzed using a Zeiss LSM 700 confocal microscope.

### Statistical analysis

The data were obtained at least in triplicate (n = 3), averaged and expressed as the mean ± standard deviation (SD). Computation of statistical significance between four types of samples was analyzed using one-way ANOVA with a post hoc Tukey’s HSD test provided by the Statsmodels module for the Python programming language.

## Electronic supplementary material

Video of the hybrid cross-linked PCL_SiHA scaffolds

Video of the hybrid well-aligned PCL_SiHA scaffolds

Video of the cross-linked PCL scaffolds

Video of the well-aligned PCL scaffolds

Supplementary Information
