# Author Correction: 3D biodegradable scaffolds of polycaprolactone with silicate-containing hydroxyapatite microparticles for bone tissue engineering: high-resolution tomography and *in vitro* study

**DOI:** 10.1038/s41598-018-35952-w

**Published:** 2018-11-29

**Authors:** Svetlana Shkarina, Roman Shkarin, Venera Weinhardt, Elizaveta Melnik, Gabriele Vacun, Petra J. Kluger, Kateryna Loza, Matthias Epple, Sergei I. Ivlev, Tilo Baumbach, Maria A. Surmeneva, Roman A. Surmenev

**Affiliations:** 1 Research Center “Physical Materials Science and Composite Materials”, National Research Tomsk Polytechnic University, 634050 Tomsk, Russian Federation; 2 Laboratory for Applications of Synchrotron Radiation, Karlsruhe Institute of Technology, Eggenstein-Leopoldshafen, Germany; 3 Institute for Applied Computer Science, Karlsruhe Institute of Technology, Karlsruhe, Germany; 4 Centre for Organismal Studies, University of Heidelberg, Heidelberg, Germany; 5 Institute for Photon Science and Synchrotron Radiation, Karlsruhe Institute of Technology, Eggenstein-Leopoldshafen, Germany; 6 Fraunhofer Institute for Interfacial Engineering and Biotechnology IGB, Stuttgart, Germany; 7 Fachbereich Chemie, Philipps-Universität Marburg, Marburg, Germany; 8 Inorganic Chemistry and Center for Nanointegration Duisburg-Essen (CeNIDE), University of Duisburg-Essen, Essen, Germany; 9 Reutlingen University, Reutlingen, Germany

Correction to: *Scientific Reports* 10.1038/s41598-018-27097-7, published online 11 June 2018

The original version of this Article contained errors. The name of Petra J. Kluger was incorrectly given as Petra Kluger.

In addition, an incorrect Affiliation was listed for Petra J. Kluger. The correct Affiliation is listed below:

Reutlingen University, Reutlingen, Germany.

These errors have now been corrected in the HTML and PDF versions of this Article, and in the accompanying Supplementary Information file.

