# Supplementary material for: 3D biodegradable scaffolds of polycaprolactone with silicate-containing hydroxyapatite microparticles for bone tissue engineering: high-resolution tomography and in vitro study
Source: Sci Rep. 2018 Jun 11;8:8907. doi: 10.1038/s41598-018-27097-7 (PMC5995873; doi:10.1038/s41598-018-27097-7)
Supplement: Supplementary file 5 — Supplementary Information [file 41598_2018_27097_MOESM5_ESM.doc]

**3D biodegradable scaffolds of polycaprolactone with silicate-containing hydroxyapatite microparticles for bone tissue engineering: high-resolution tomography and *in vitro* study**

**Svetlana Shkarina^1,5^, Roman Shkarin^2,3^, Venera Weinhardt^2,4,5^, Elizaveta Melnik^1^, Gabriele Vacun^6^, Petra J. Kluger^9^, Kateryna Loza^8^, Matthias Epple^8^, Sergei I. Ivlev^7^, Tilo Baumbach^2,5^, Maria A. Surmeneva^1,*,+^, Roman A. Surmenev^1,*,+^**

^1^Research Center "Physical Materials Science and Composite Materials", National Research Tomsk Polytechnic University, 634050, Russian Federation

^2^Laboratory for Applications of Synchrotron Radiation, Karlsruhe Institute of Technology, Eggenstein-Leopoldshafen, Germany

^3^Institute for Applied Computer Science, Karlsruhe Institute of Technology, Karlsruhe, Germany

^4^Centre for Organismal Studies, University of Heidelberg, Heidelberg, Germany

^5^Institute for Photon Science and Synchrotron Radiation, Karlsruhe Institute of Technology, Eggenstein-Leopoldshafen, Germany

^6^Fraunhofer Institute for Interfacial Engineering and Biotechnology

IGB, Stuttgart, Germany

^7^Fachbereich Chemie, Philipps-Universität Marburg, Marburg, Germany

^8^ Inorganic Chemistry and Center for Nanointegration Duisburg-Essen (CeNIDE), University of Duisburg-Essen, Essen, Germany

^9^ Reutlingen University, Reutlingen, Germany


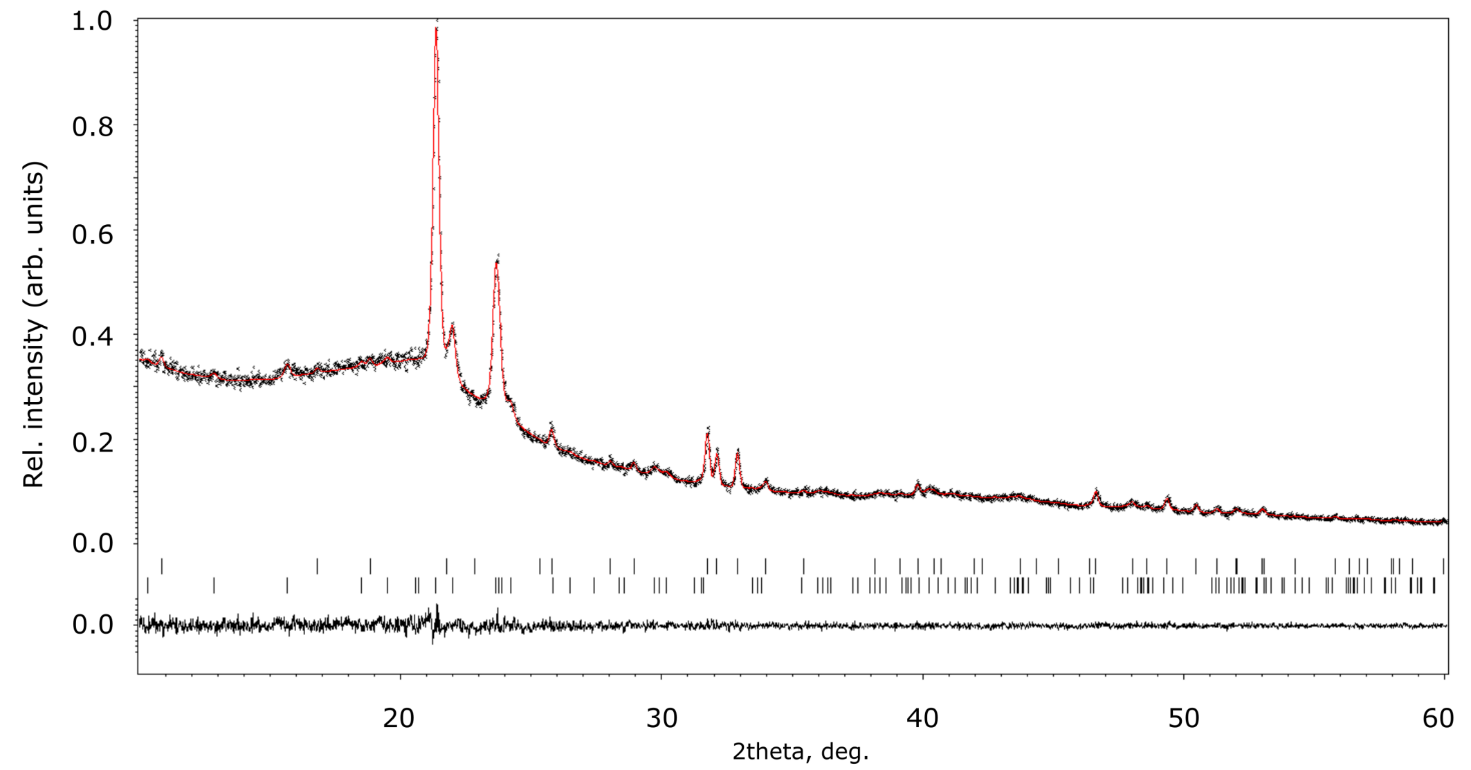


**Figure S1**. Powder X-ray diffraction pattern of PCL-SiHA at 293 K. Shown are experimental data (black crosses), Le Bail profile (red), and differential profile (bottom, black). Calculated reflection positions are shown as black ticks (first row for SiHA, second row for PCL). Rp = 0.0232, wRp = 0.0302.


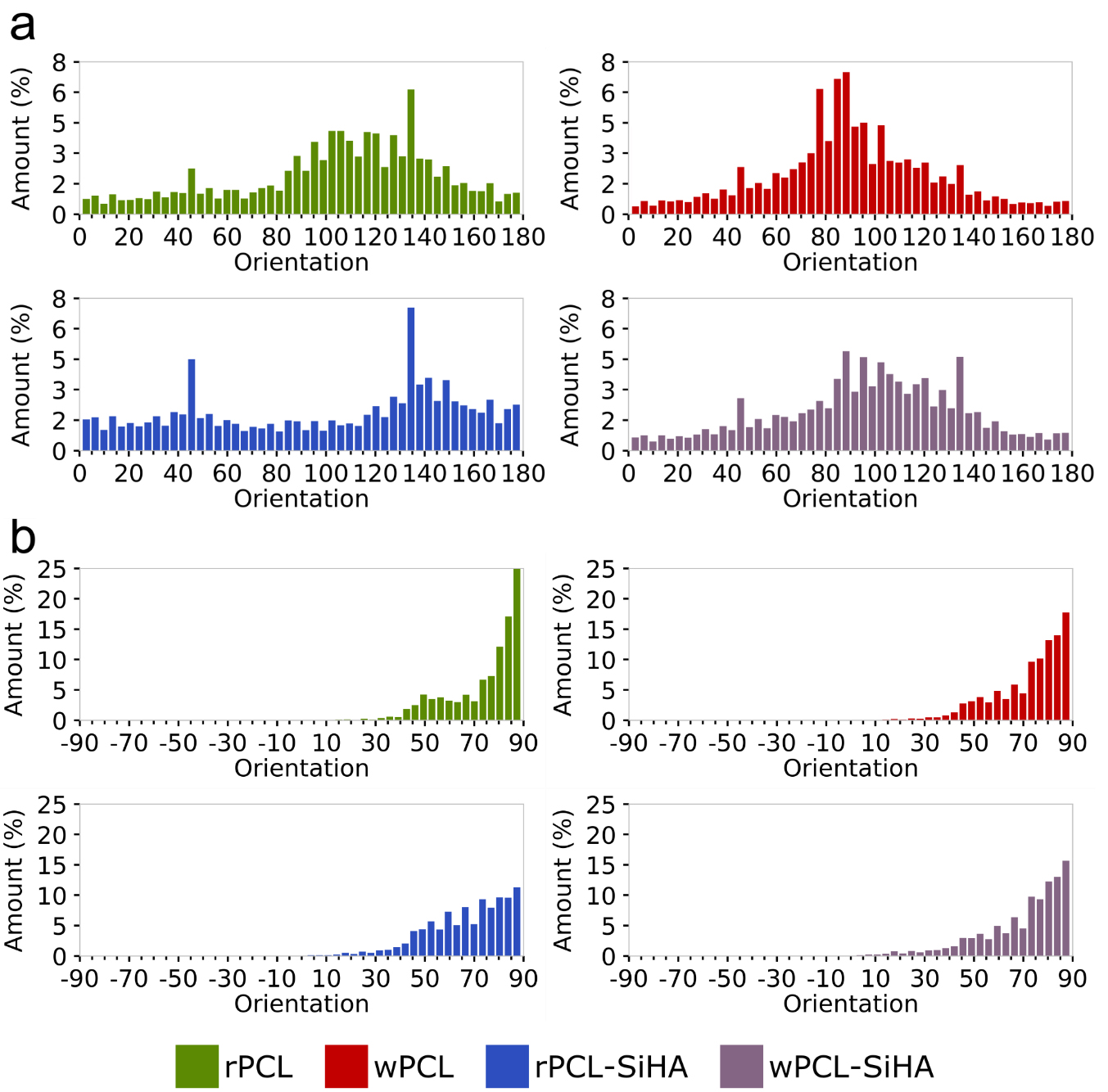


**Figure S2**. X-ray µCT-based analysis of (a) azimuthal and (b) latitudinal fiber orientation calculated for 3D scaffolds.


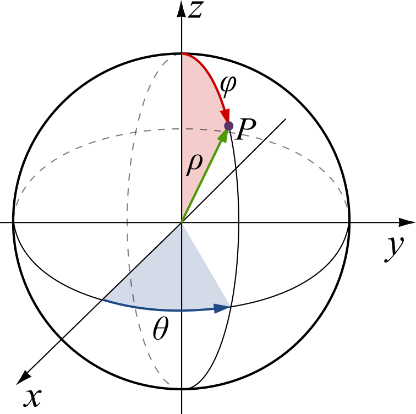


**Figure S3**. Diagram of a laboratory coordinate system in *x*, *y*, and *z*. A fiber begins at the center of the coordinate system and ends at point *P*; this can be represented by a vector *ρ* with azimuth and latitude angles *θ* and *φ*, respectively.
